# Geochemical Behaviors of Rare Earth Elements (REEs) in Karst Soils under Different Land-Use Types: A Case in Yinjiang Karst Catchment, Southwest China

**DOI:** 10.3390/ijerph18020502

**Published:** 2021-01-09

**Authors:** Ruiyin Han, Zhifang Xu

**Affiliations:** 1 Key Laboratory of Cenozoic Geology and Environment, Institute of Geology and Geophysics, Chinese Academy of Sciences, Beijing 100029, China; ruiyinhan@163.com; 2 The College of Resources and Environmental Engineering, Guizhou University, Guiyang 550025, China; 3 CAS Center for Excellence in Life and Paleoenvironment, Beijing 100044, China; 4 University of Chinese Academy of Sciences, Beijing 100049, China

**Keywords:** rare earth elements (REEs), land-use types, soil profile, karst catchment, southwest China

## Abstract

The geochemical characteristics of rare earth elements (REEs) can be employed to identify the anthropogenic and natural influence on the distributions of REEs in soils. A total of 47 soil samples from the three soil profiles of the secondary forest land, abandoned cropland, and shrubland in the Yinjiang county of Guizhou province, southwest China, were collected to determine the contents and distribution of REEs in the soil environment. The total REEs (ΣREE) contents in different soil profiles are in the following sequence: secondary forest land (mean: 204.59 mg·kg^−1^) > abandoned cropland (mean: 186.67 mg·kg^−1^) > shrubland (mean: 139.50 mg·kg^−1^). The ratios of (La/Gd)_N_ and (Gd/Yb)_N_ ranged from 0.62 to 1.00 and 1.18 to 2.16, which indicated that the enrichment of the medium rare earth elements (MREEs) was more obvious than that of the light rare earth elements (LREEs) and the heavy rare earth elements (HREEs). The phenomenon could be attributed to the preferential absorption of MREEs by fine particles and the substitution of Ca^2+^ by MREEs. Most soil samples were characterized by the negative Ce anomalies (anomalies values: 0.30–1.10) and positive Eu anomalies (anomalies values: 0.43–2.90). The contents of REEs in the profiles of secondary forest land and shrubland were mainly regulated by soil pH and Fe contents while clay content and agricultural activities were the main controlling factors in the soil profile of abandoned cropland. This study highlights the role of agricultural activities in affecting the distributions of REEs in karst soils, which could provide some insights for the protection of the soil environment.

## 1. Introduction

Rare earth elements (REEs) usually comprise the 15 lanthanide elements from La to Lu, and these elements are similar in chemical properties [1,2]. Based on the atomic weight, REEs can be divided into the light rare earth elements (LREEs, from La to Sm), the medium rare earth elements (MREEs, from Eu to Dy), and the heavy rare earth elements (HREEs, from Ho to Lu) [3,4]. The fractionation of REEs is usually accompanied by the variations of environmental parameters due to the stable chemical properties of REEs in the soil environment [5]. For example, Ce and Eu are normally presented as trivalent ions in soils, but they can also be oxidized or reduced into strongly active Ce^4+^ or Eu^2+^ with the changes in redox conditions [6,7,8]. Therefore, the great Ce and Eu anomalies always occur with the increasing chemical activities, which can be used to denote the changes in soil environment reversely. In the last century, REEs have been widely used in fertilizers and pesticides in agricultural activities to obtain high-yield and high-quality crop [9]. Exogenous input can interfere with the geochemical behaviors of REEs (such as the enrichment or the fractionation of REEs) under natural conditions [10,11]. Therefore, distinguishing anthropogenic sources from natural sources benefits the accurate estimation of soil pollution.

In the last 50 years, the distribution and fractionation of REEs have been employed to identify the weathering and ecological effects and indicate the changes of local soil environment [12,13,14,15,16,17,18,19]. Many scholars have reported the distribution, fractionation, and other behavioral characteristics of REEs in different types of soils (clayey, sandy, peaty, and other soils) and different geological environments (limestone, granite, and carbonatite) [10,20,21,22]. Fully understanding the geochemical behaviors of REEs in the soils under different environmental conditions can help to predict the migration and transformation processes of REEs in the soils [23,24,25]. Besides the natural processes, anthropogenic inputs, such as industrial, agricultural and mining activities, also affect the distribution and fractionation of REEs in the horizontal and vertical directions of soils, of which the agricultural input is the most prominent [11,26,27]. The geochemical behavior of REEs in many types of soil profiles and the influence of human activities on the processes of weathering and the origin of the rocks have been widely studied previously [6,9,20,28,29]. However, the geochemical characteristics of soil REEs in different types of lands, especially in karst areas, are rarely investigated. Understanding the sources, distribution, migration, and transformation of REEs is crucial for the environment management in karst regions [9].

Yinjiang county is a typical karst region in the east of Guizhou province, southwest China, with a population of about 454,800. The unique geological environment of the karst region makes the soils very susceptible to human activities [30]. Yinjiang county is an important agricultural region where agricultural activities are the main sources of exogenous REEs. Analyzing the geochemical behavior of REEs in karst areas helps to understand the migration and fractionation of REEs in soils. Furthermore, it is helpful to judge the effects of human activities on soil formation processes. In the present study, the distribution of REEs in the soil profiles of the secondary forest land, abandoned cropland, and shrubland were analyzed: (1) to explore the geochemical behavior of REEs in the profiles under different land-use types; (2) to determine the influence of natural processes and human activities on the distribution, migration and transformation of REEs in the soils; (3) to investigate the environmental implications of soil REEs in karst areas.

## 2. Materials and Methods

### 2.1. Study Area

**Figure 1:**
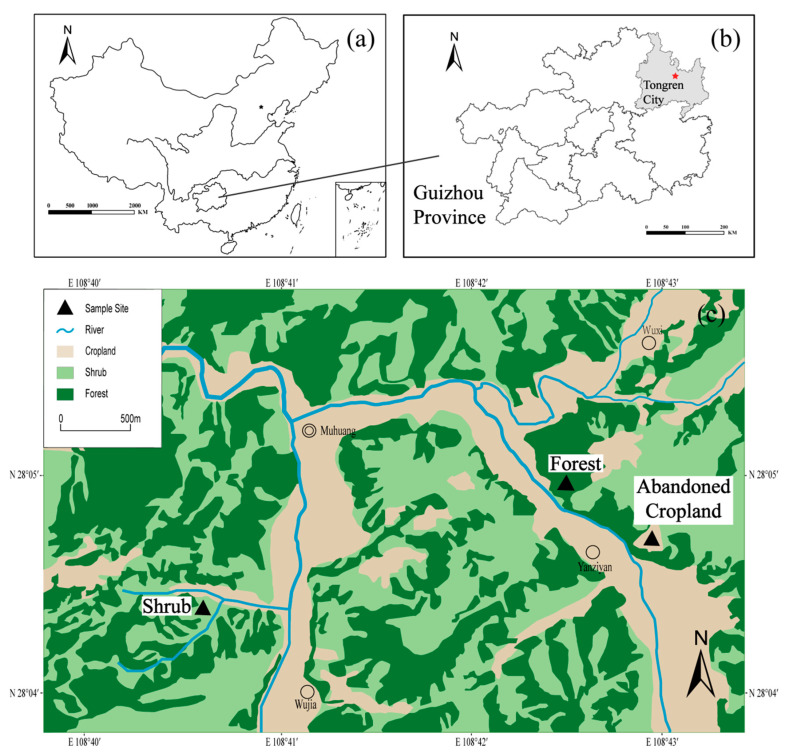
Distribution of land-use types and sample sites in Yinjiang catchment, southwest China. Maps of China (**a**) and Guizhou province (**b**).

The study area was located in Yinjiang Tujia and Miao Autonomous County, the west of Tongren city in the northeast of Guizhou province (27°35′19″–28°20′32″ N, 108°17′52″–108°48′18″ E), southeast China (Figure 1) [31]. Yinjiang county is dominated by the subtropical monsoon climate with the annual precipitation of about 1100 mm and the average air temperature of 16.8 °C [32]. The rainy season is usually from April to October [33]. Agriculture has been the main economic source in Yinjiang county for a long time due to the limitations of natural conditions (terrain, rivers, karst region), and the development of industries has also been initiated over these years [34]. According to the statistical results in 2016, the forest coverage rate of Yinjiang county was 64.71%, 93.29% of which is the mountainous, and the farmland area occupied more than 25% of the total area in Yinjiang county. The average altitude is more than 1600 m, and the elevation decreases from the east to the west with a maximum altitude difference of 2116.1 m [35,36]. The study basin is a typical karst trough valley area [37], where soils were mainly derived from Ordovician carbonate rocks [38]. There are 7 types of soils across the whole Yinjiang catchment, including the yellow soils, yellow-brown soils, lime soils, and so on. The distribution of land-use types in this region is shown in Figure 1. The detailed information of the sampling sites and soil profiles is given in Table 1.

### 2.2. Soil Sampling and Analysis

The sample sites were far away from the mining operations and residential areas. The applications of fertilizers and pesticides in agricultural lands were the main sources of exogenous REEs of soils [34]. In September 2016, about 1 × 1 m pit were dug at the three sits under diverse land-use types, including secondary forest land (T1), abandoned cropland (T2) and shrubland (T3). The three samples at same depth from three sides of the pit were collect, then mixed to be one sample. A total of 47 soil samples (T1, *n* = 20; T2, *n* = 16; T3, *n* = 11) were collected for the analyses of physiochemical parameters and REEs contents. The influence of human activities is limited in forest land, weak in the abandoned cropland that has been converted from cropland where abundant fertilizer and compost have been applied but strong in the shrubland due to the occasional grazing. Giving livestock may affect the physical structure and alter the chemical composition of soil [39]. The land use types and elevations of the three soil profiles are shown in Figure 1 and Table 1. Soil samples were collected at the 5 cm interval at the depth of 0–20 cm while the left samples were collected at the 10 cm interval.

After the removal of roots and stones, the samples were dried at room temperature. Then, the dried samples were fully grounded until all of them filtered through a 200-mesh sieve. In addition, 10% HCl and 30% H_2_O_2_ were applied to eliminate the organic bonding agents and calcareous cement before the percentages of clay, silt, and sand of soil samples were determined by the laser particle analyzer (Mastersizer2000, Malvern, UK), with the precision of 1%. After soil solutions (soil:water = 1:2.5) were well prepared and left to stand for 30 min, soil pH was determined by a pH-meter whose precision was ±0.05. All soil samples were digested with the mixture of HNO_3_-HF-HClO_4_ [40,41,42]. Around 50 mg of the powder samples were digested with 3 mL HNO_3_, 3 mL HF, and 1 mL HClO_4_ in Teflon beakers. Then, samples were heated on a hotplate at 120° for 3 days. After digestion, samples were heated and evaporated to about 0.5 mL, then diluted with ultra-pure water to 25 mL. The digestions of soil samples were committed in the Laboratory of Surficial Environmental Geochemistry, CUGB. The contents of REEs and other major elements were respectively analyzed by ICP-MS (Elan DRC-e, Perkin Elmer) (REEs) and ICP-OES (Optima 5300DV, Perkin Elmer, US) (Fe and Mn), with the precision better than ±0.01 g·kg^–1^ for REEs and less than ±0.02 mg·kg^–1^ for Fe and Mn. Quality control and quality assurance were performed by the procedural blank and standard reference material (GBW07447 and GBW07449) in the Institute of Geographic Sciences and Natural Resources Research, CAS.

### 2.3. Data Analysis

The contents of REEs in the soils confirm to the rule of Oddo-Harkins. To eliminate the “Parity effect” and observe the fractionation of REEs more clearly, the REE data of samples were normalized by the Post-Archaean Australian Shale (PAAS) [43].

The majority of REEs usually occur as positive trivalent ions in natural environmental systems. However, due to the different atomic structures, some elements can also exist in the form of positive tetravalent and positive divalent ions. For instance, Ce^3+^ can be oxidized to Ce^4+^ and Eu^3+^ can be reduced to Eu^2+^, which makes them more fractionated relative to the neighbor elements [5]. The formulas to calculate the Ce and Eu anomalies are shown as follows [44,45]:

$$\delta \mathrm{Ce}\;=Ce_{N}/{\left(La_{N}\times Pr_{N}\right)}^{0.5}\;\;\delta \mathrm{Eu}\;=Eu_{N}/{\left(Sm_{N}\times Gd_{N}\right)}^{0.5}$$

where REE_N_ represents the PAAS-normalized contents. The δCe and δEu values higher than 1 denote positive anomalies while those lower than 1 denote negative anomalies.

Enrichment factor (EF) can represent the enrichment degree of the specific element relative to the reference element (weak mobility) in soils and helps to distinguish the sources of elements [46]. The elements Cr, Mn, Fe, Zn, Cu, Sb, Hg and Al have been widely employed as the reference elements [47,48,49]. The EF_REE_ is defined as follows [48,50]:

$$EF_{REE}={\left(\left[REEs\right]/\left[Al\right]\right)}_{S}/{\left(\left[REEs\right]/\left[Al\right]\right)}_{UCC}$$

where *S* implies soil samples, and *UCC* implies the upper continental crust. The contents of Al and REEs in the upper continental crust are reported by Taylor et al. [51].

Origin 2017 (OriginLab, Northampton, UK) and Statistics software package of SPSS 25.0 (IBM SPSS Statistics, Chicago, IL, USA) were used for graphics drawing and data analyses.

## 3. Results and Discussion

### 3.1. Distributions of REEs in Soil Profiles

**Figure 2:**
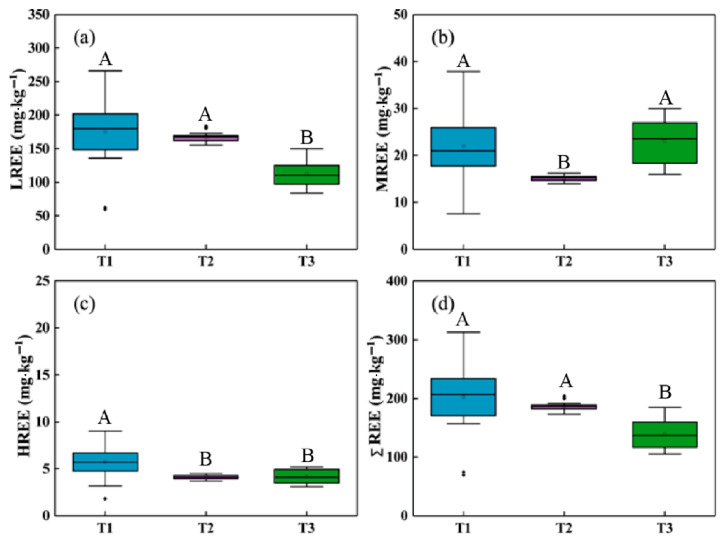
Box diagrams of the contents of REEs in three profiles, (**a**) LREE; (**b**) MREE; (**c**) HREE; (**d**) ΣREE. T1: secondary forest land; T2: abandoned cropland; T3: shrubland. Different uppercase letters indicate significant differences in LREE, MREE, HREE, and ΣREE contents between the T1, T2, and T3 profiles, based on the one-way ANOVA with the LSD test at the level of *p* < 0.05.

The contents of total REEs (ΣREE), LREEs, MREEs, and HREEs in the three soil profiles are shown in Figure 2, and those values are listed in Table A1. The average ΣREE contents of the T1, T2, and T3 profiles were 202.86 mg·kg^−1^, 186.67 mg·kg^−1^, and 139.50 mg·kg^−1^, respectively. The difference of average ΣREE contents is significant between T1 and T3, T2, and T3 profiles at the 0.05 significance level while that between T1 and T2 profiles is not significant. The content of ΣREE in the soils under secondary forest land (T1) is the highest. The contents of ΣREE in T1 and T2 profiles are higher than those of Earth’s crust (153.80 mg·kg^−1^) and the average level in Chinese soils (176.76 mg·kg^−1^) [10,52].

The ΣREE content is the highest in the surface soil of the secondary forest land (T1), which is likely associated with the abundant dead leaves and plant roots. The enrichment of REEs in the processes of plant growth and decay can lead to the increasing ΣREE in the surface soils [53]. The organic matter contents of forest surface soil are generally higher than those of other types of soils, and REEs can be strongly complexed by the organic matter under neutral pH, which also improves the stability of REEs [54,55]. Generally, the content of soil organic carbon (SOC) in abandoned cropland is low, but it can increase slowly in a short time once the abandoned cultivation period is over [56,57,58,59]. The content of ΣREE was almost constant in the soil profile of the abandoned cropland (T2), which is possibly associated with the distributions of clay particles and SOC in profile. The potential mobility of REEs is quite low because REEs are usually absorbed by the clay minerals and organic matter in abandoned cropland [60,61]. Generally, SOC content decreases with increasing soil depth [62,63]. The T2 profile was located in an abandoned cropland that has experienced 50-year agricultural activities and a 3-year abandoned period. The SOC content in the surface soils of abandoned cropland is lower than that in the soils covered by normal vegetation because of the limited SOC input from surface vegetations [64]. Thus, the distributions of REEs in the T2 profile were not significantly affected by the low SOC content. Moreover, the external REEs were possibly leached to the bottom of the soil profile during the 50-year abandoned period. The REEs contents in the T2 profile were higher than those in the T3 profile, which may be attributed to the external inputs of agricultural activities. The five-year moderate grazing on the shrubland might not exert much influence on the REEs contents and distributions in the profile T3.

**Figure 3:**
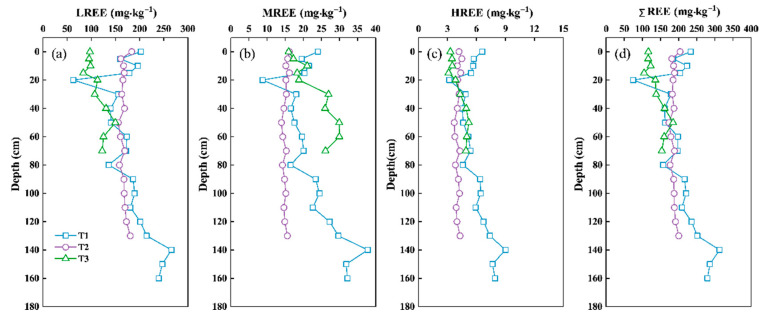
Vertical distributions of the contents of REEs in three profiles, (**a**) LREE; (**b**) MREE; (**c**) HREE; (**d**) ΣREE. T1: secondary forest land; T2: abandoned cropland; T3: shrubland.

The LREE contents account for a large proportion of ΣREE contents in the three profiles, which is in line with the characteristics of REEs in the soils of Guizhou Province [53]. Our results show that LREE content was the highest (>77%), followed by MREE, and then the HREE (Figure 3). In the humid karst region, abundant inorganic ions (e.g., HCO_3_^−^, CO_3_^2−^) would be produced in strong chemical weathering processes under neutral to alkaline conditions. These ions prefer to complex with HREEs rather than LREEs in the neutral to alkaline solution, which accounts for the greater solubility and mobility of HREEs and MREEs [65,66].

Overall, the REEs contents tend to increase with soil depth in spite of a positive peak in the soil horizons of 20–90 cm of the three soil profiles, which is related to the leaching loss during soil development [67]. Yellow and brown soils are distributed in the upper horizon while brown and red soils are presented in the lower horizon. It is reported that the REEs contents in the red soils are larger than those in loess and brown soils [53]. The REEs content in loess was the lowest in the present study. The bottom of the T1 profile was the weathering crust of carbonate rocks, and the content of REEs in the weathering crust was obviously lower than that in upper soils. The variation of REEs contents in the T2 profile was different from that in the other two profiles, which may be induced by the soil erosion and tillage disturbance during the 50 years of agricultural activities.

### 3.2. The Fractionation Patterns of LREE, MREE, and HREE

**Figure 4:**
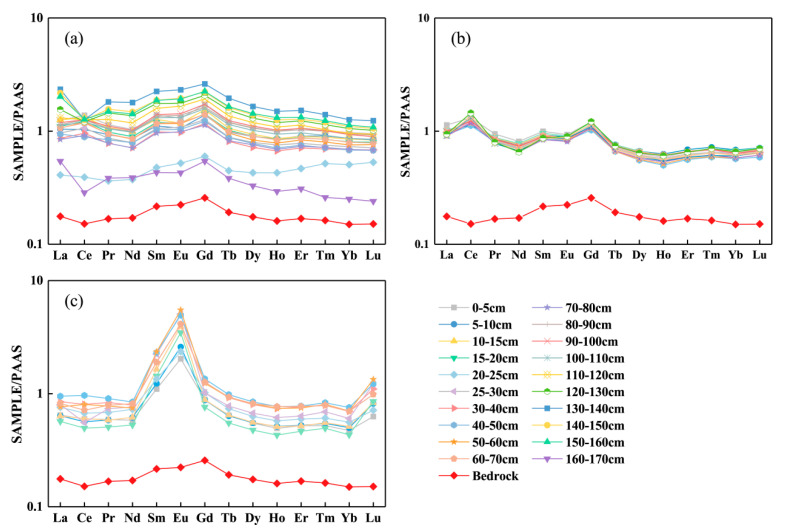
REEs fractionation patterns (PAAS- normalized) in three soil profiles, (**a**) T1 profile, (**b**) T2 profile, (**c**) T3 profile. (The mean normalized REEs of the bedrock of T1 and T3 profile were adopted as those of T2 profile because the bedrock samples were unavailable in T2 profile).

The relative abundance of REEs (i.e., fractionation) would change when it was influenced by soil pH, temperature, humidity, soil properties, and other environmental parameters [68]. As shown in Figure 4, the distribution patterns of REEs in the T1 and T2 profiles were similar to those in the bedrock, in contrast, the MREEs were severely enriched in T3 profile, which might be dominated by other factors. The ratios of (La/Yb)_N_, (La/Gd)_N_, and (Gd/Yb)_N_ can denote the fractionation among LREE, MREE, and HREE, where N means normalized value with PAAS [60]. The (La/Yb)_N_, (La/Gd)_N_, and (Gd/Yb)_N_ values are shown in Table 2.

The calculation displayed that the ratios of (La/Gd)_N_ in all soil samples were in the range from 0.62 to 1.00 and most of them (>90%) were less than 0.90. The ratios of (La/Yb)_N_ in most soil samples were larger than 1 (only one was less than 1), ranging from 0.81 to 2.16. The ratios of (Gd/Yb)_N_ in all soil samples were higher than 1 and in the range of 1.18–2.16. The results showed that the fractionation of MREEs was stronger than that of LREEs and HREEs in most soils. This phenomenon could be attributed to the following potential factors: (1) MREEs can preferentially complex with high molecular organic compounds [69]; (2) the absorption of MREEs by particulate matter is greater than that of LREEs and HREEs under the combined influence of REEs hydrolysis degree and free ionic concentration variation [70]. The slight relative enrichment of HREEs to LREEs is attributed to the strong compatibility between them and anions such as HCO_3_^−^, CO_3_^2−^, and Cl^−^ [71].

### 3.3. Ce and Eu Anomalies in Karst Soils

#### 3.3.1. Ce Anomalies

The Ce anomaly values (δCe) are shown in Table 2. The soils in three profiles show evidently negative Ce anomalies. The δCe values ranged from 0.30 to 0.89 in the T1 profile, from 0.85 to 1.10 in the T2 profile and from 0.27 to 0.71 in the T3 profile. The migration rate of Ce in red soils and loess is high [65]. In the present study, the δCe values in brown-red soils were greater than 1.

The δCe values can be used to reflect the soil redox environment. Generally, Ce^3+^ is easily oxidized to Ce^4+^ and absorbed by hydroxide, Mn-oxide, and Fe-oxide [72,73,74]. A large amount of REEs is released into soils during the rock weathering processes while Ce^4+^ remains in the regolith, resulting in the depletion of Ce in soils. This process often occurs in the region of carbonate rocks [29]. In addition, Ce^4+^ can form stable soluble compounds and be filtered by alkaline media (i.e., CO_3_^2−^ and HCO_3_^−^) [75].

#### 3.3.2. Eu Anomalies

The values of Eu anomalies (δEu) are shown in Table 2. The δEu values ranged from 0.43 to 1.05 in the T1 profile, from 0.59 to 0.62 in the T2 profile, and from 1.53 to 2.90 in the T3 profile. The results indicated that the soil samples from the T1 and T2 profiles showed negative Eu anomalies while those from the T3 profile showed obviously positive Eu anomalies. In the reducing environment, Eu^3+^ can be reduced to Eu^2+^, resulting in the separation of Eu from other REEs [76,77]. Similar to the geochemical behavior of Sr^2+^, Eu^2+^ is also easily leached in soil profiles [78]. The depletion of Eu in the T1 and T2 profiles is consistent with their bedrock, which might be inherited from the bedrock. However, the positive Eu anomalies presented in the T3 profile were opposite to those in the bedrock, which could be explained by the fact that Eu^2+^ can co-precipitate with Ba^2+^ to form insoluble sulfates and accumulate in soils [79]. Guichard et al. [80] considered that the positive Eu anomalies in barite might be attributed to the replacement of Ba^2+^ by Eu^2+^ in the reducing environment. Similarly, Ca^2+^ in feldspar minerals may also be substituted by Eu^2+^, resulting in the positive Eu anomaly [64]. It can be seen that bedrock may be the main source of Eu in the T1 and T2 profiles, while the soil processes had a stronger effect on the Eu fractionation in the T3 profile.

Besides the migration, numerous studies have shown that the changes in redox conditions have a great influence on Ce and Eu anomalies [22,29,81,82]. In the oxidation environment, the values of δCe and δEu are less than 1, while they are greater than 1 in the reduction environment [83]. Thus, the soils in the T3 profile may be in a reducing environment, while the soils in the T1 and T2 profiles are in an oxidizing environment.

### 3.4. Impacts of Soil Properties on REEs Distribution

**Figure 5:**
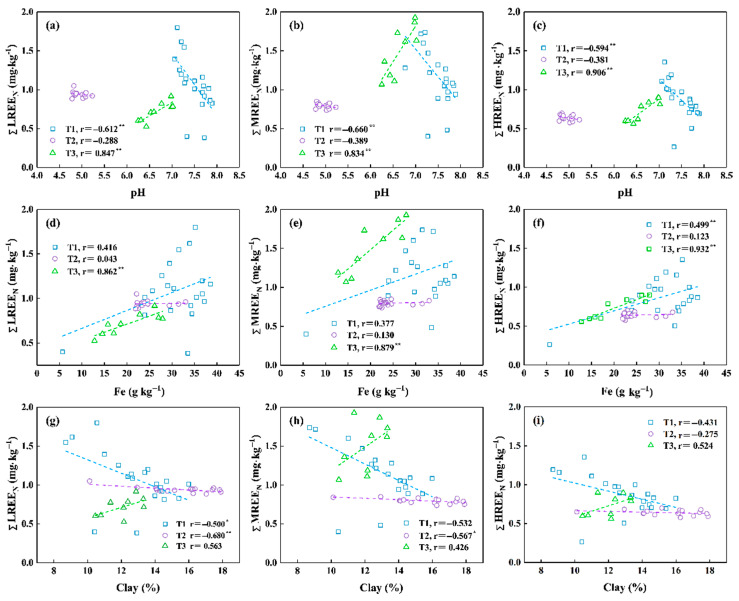
Relationships between pH, Fe content, and clay content and ∑REE content in the T1 to T3 soil profiles under diverse land-use types. (* *p <* 0.05; ** *p <* 0.01). LREE–pH (**a**), MREE–pH (**b**), HREE–pH (**c**), LREE–Fe (**d**), MREE–Fe (**e**), HREE–Fe (**f**), LREE–Clay (**g**), MREE–Clay (**h**), HREE–Clay (**i**).

In addition to the structure of REEs and soil redox environment, the distributions of REEs are affected by many soil properties, including soil pH, clay content, Fe content, and so on. The relationships between these soil properties and REEs contents are shown in Figure 5.

#### 3.4.1. Soil pH

The soil pH ranged from 6.77 to 7.89 in the T1 profile, from 4.78 to 5.23 in the T2 profile and from 6.25 to 7.02 in the T3 profile. The soil pH values of the profile T2 were much lower than those of the other soil profiles because agricultural soils were always acidified (pH < 6) during the cultivation period [84]. The hydrolytic strength and activity of REEs increase with the pH values [5,52]. The hydrolysis strength of LREEs is stronger than that of MREEs and HREEs, but their activities are opposite [85,86]. REEs tend to form stable complexes with HCO_3_^−^ in alkaline environment because high pH would promote the activity of HCO_3_^−^ [4]. Soil pH positively correlated with REEs content in the T3 profile but negatively correlated with it in the T1 profile (Figure 5). According to Song’s experiment [87], when soil pH < 5, the hydrolysis rate of most REEs (except La^+^, Ce^+^ and Nd^+^) is less than 10%; when pH > 6, only a few REEs show obvious hydrolysis; when pH > 7, the degree of hydrolysis rate is obviously improved. The pH values in the soil profile T2 were generally less than 5.1. It was inferred that the hydrolysis rate of REEs in the T2 profile was low. The positive correlation between REEs contents and soil pH in T2 profile may be related to the adsorption of negatively charged groups by particulate matter. The higher contents of OH^−^ and other negative groups are more easy to combine with the dissolved REEs in the alkaline environment [66]. The values of pH were almost greater than 7 in the soil profileT1, and the adsorption rate decreased because the hydrolysis of REEs is strong when the soil pH ranges from 7 to 9 [87]. However, Figure 5 shows that pH and REEs content in T2 profile are not correlated, which may result from other factors.

#### 3.4.2. Clay Content

Clay minerals are important in affecting the distribution of REEs because they are the major carriers of the REEs of adsorption state. Clay contents ranged from 8.71–15.99% in the T1 profile, 10.12–17.90% in the T2 profile, and 10.45–13.33% in the T3 profile. Clay contents were negatively correlated with the contents of LREE, MREE in the T2 profile while only the negative correlation with the LREE contents was observed in the T1 profile, and the correlation between clay contents and REEs contents was not significant in the T3 profile (Figure 5). The positive correlation between clay contents with REEs contents (especially the LREE contents) was widely reported in many studies [86,88,89,90]. While the REEs contents were negatively correlated with clay mineral contents in the T1 and T2 profiles, it cannot completely explain the behaviors of REEs.

#### 3.4.3. Fe Content

The Fe contents were in the range from 5.68 g·kg^−1^ to 38.56 g·kg^−1^ in the T1 profile, from 21.92 g·kg^−1^ to 33.92 g·kg^−1^ in the T2 profile, and from 12.80 g·kg^−1^ to 27.93 g·kg^−1^ in the T3 profile (Figure 5). REEs can be absorbed by Fe oxides, thus REEs contents generally closely correlate with Fe contents. The contents of REEs were closely associated with Fe contents in the soil profile T3. Ran and Liu [91] demonstrated that Fe oxides can perform obligate adsorption on REEs, and the adsorption capacity would increase with the pH. The content of Fe oxides also increases with soil evolution, and high REEs contents are often found in the red soils, Southern China [92].

### 3.5. Environmental Effect of REEs

#### 3.5.1. Enrichment Factors (EFs) of REEs

**Figure 6:**
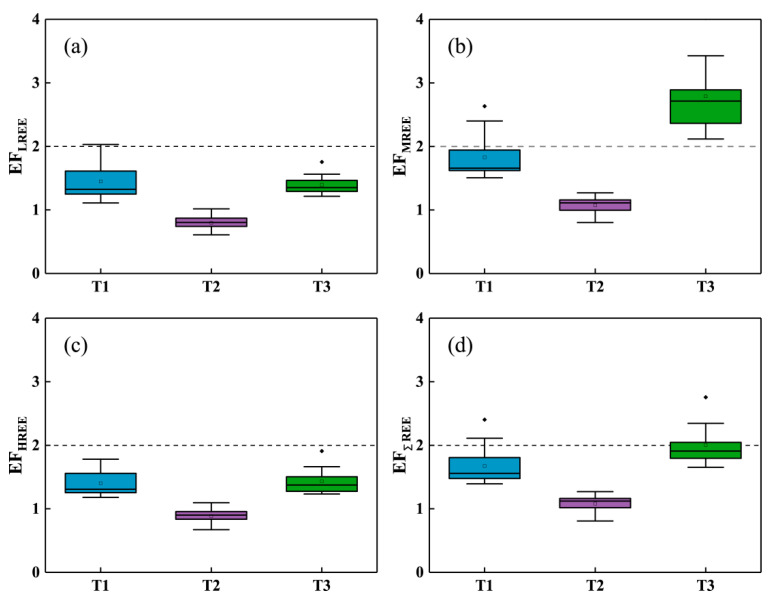
Boxplots of the EFs for REEs contents in three profiles, (**a**) LREE; (**b**) MREE; (**c**) HREE; (**d**) ΣREE. T1: secondary forest land; T2: abandoned cropland; T3: shrubland.

Enrichment Factors (EFs) have been frequently applied to evaluate the enrichment degree of elements in soils and to identify external input sources [46]. According to the EFs index, REEs enrichment degree can be divided into micro enrichment (EF < 2), medium enrichment (2 < EF < 5) and heavy enrichment (EF > 5), of which the higher EF values denote more anthropogenic input [93]. In addition to the MREE in the soils of the T3 profile, the EFs of ΣREE, LREE, MREE, and HREE in most soils of the three profiles are lower than 2 (Figure 6). The result denoted that the REEs in the studied soil profiles were mainly derived from the weathering processes of the parent rocks. The enrichment of MREE in the T3 profile mainly leads to Eu anomaly, as analyzed above. The EF > 2 values were only found in the brown–red soils from 120 cm to 160 cm in the T1 profile. Several studies attributed the higher REEs contents in brown–red soils to the appropriate hydrothermal conditions for the enrichment of REEs [53,88].

#### 3.5.2. Environmental Implication of Soil REEs

The migration processes of REEs in soils of different land–use types are various. Due to the different intensities of anthropogenic influence, the distribution and fractionation of REEs under these land–use types are different in the study area. The contents of REEs in the soils are mainly controlled by the geological background of the area, but the sources of REEs are also related to weathering and external disturbance.

The enrichment of REEs in the abandoned cropland profile is different from that in the soil covered by normal vegetation. The results confirmed that the agricultural activities were essential in regulating the geochemical behavior of REEs. Comparing the REEs contents in the farmland with those in the abandoned cropland in Puding county, REEs contents were higher in the shallow and deep soil horizons but relatively low in the middle horizon, which could be attributed to the external input from agricultural activities and leaching processes [60]. However, after the abandonment of farmland, the REEs contents in the soils decreased. The REEs contents in the abandoned cropland in the study area were almost constant along with soil depth, and the values of EF were below 1.27, which might result from the reduction of SOC contents during the period of abandonment. The contents of REEs in the surface soil of shrubland were low, while those in deeper horizons were not much different from those in secondary forest and abandoned cropland. This may be related to the goat herding activities in the past five years. There are many agriculture–based karst areas, of which the Yinjiang county is one typical region. Exploring the distribution and fractionation characteristics of soil REEs in karst areas helps to understand the geochemical behavior of REEs in the specific soil environment, which can provide statistical support for the agricultural production and restoration of abandoned cropland.

## 4. Conclusions

The REE’s distribution and influence factors were distinctive in the three profiles of secondary forest land, abandoned cropland and shrubland in Yinjiang catchment. The ΣREE contents were the highest in the secondary forest land profile and lowest in the shrubland profile. The ΣREE contents in all soil samples were higher than those in bedrocks, indicating that although weathering is a main controlling factor of REEs, the distribution and fractionation of REEs (especially Ce and Eu anomalies) are affected by the changes of soil properties and redox environment under subsequent pedogenesis. The fractionation of MREE is more obvious than that of LREE and HREE in most soils because MREE are more easily absorbed by clay particles and complexed by organic compounds.

Meanwhile, agricultural activities also exert an influence on the distributions of soil REEs. The contents of REEs in the profiles of the secondary forest land shrubland profile are mainly dominated by natural factors, including organic matter, Fe oxides, and so on. However, the influence factors of REEs in the abandoned cropland profile are more complex. The hydrolysis rate of REEs in the abandoned cropland was low and the clay contents were negatively correlated with the contents of REEs. In addition to organic matter, anthropogenic input may also affect the distribution of REEs in the abandoned cropland.

## Figures and Tables

**Table 1:** General descriptions of three soil profiles in the study area.

Land-Use Types	Location	Elevation (m)	The Situation of Land Use	Soil Profile Characteristics
Secondary forest land (T1)	28°04′57.64″ N 108°42′31.01″ E	838	Subtropical evergreen broad-leaved secondary forest.	0 to 20 cm: The surface layer is gray soil with more plant roots and dead leaves. The deep is yellow soil with a few small stone particles. 20 to 110 cm: The upper part is yellow with a few stones. 110 to 160 cm: The lower part is brown-red soil with almost no stones.
Abandoned cropland (T2)	28°04′48.35″ N 108°42′58.22″ E	892	Sloping farmland. Surrounded by subtropical evergreen secondary forests, the area has been cultivated for about 50 years, and has been abandoned for three years before sampling.	0 to 20 cm: Yellow soil with a few small stone particles and plant roots. 20 cm to 120 cm: The upper part is yellow and brown soil. 120 cm to 130 cm: The lower part is brown-red soil with more stones.
Shrubland (T3)	28°04′22.68″ N 108°40′37.62″ E	776	Native shrub grass. The soil layer is relatively shallow, and some bedrock is exposed. In the last 5 years, a small number of goats were released here.	0 to 20 cm: The upper part is black soil with more humus and a few stones, while the lower part is gray soil with more stones. 20 cm to 70 cm: Yellow soil with a few stones.

**Table 2:** The information of REE in soil profiles.

Sample Sites	Depth (cm)	∑REE (mg·kg^−1^)	(La/Gd)_N_	(La/Yb)_N_	(Gd/Yb)_N_	δCe	δEu
T1	0	233.14	0.72	1.28	1.77	0.89	0.74
	5	184.71	0.75	1.21	1.61	0.74	0.67
	10	223.40	0.81	1.47	1.81	0.87	0.70
	15	204.29	0.78	1.37	1.76	0.85	0.73
	20	74.58	0.69	0.81	1.18	0.44	0.50
	30	170.01	0.75	1.29	1.72	0.77	0.69
	40	162.76	0.77	1.38	1.80	0.67	0.69
	50	198.18	0.75	1.41	1.87	0.85	0.73
	60	197.57	0.76	1.34	1.76	0.83	0.75
	70	157.06	0.75	1.23	1.64	0.72	0.68
	80	216.44	0.70	1.21	1.72	0.85	0.80
	90	220.56	0.69	1.27	1.85	0.83	0.81
	100	209.39	0.71	1.30	1.83	0.81	0.77
	110	235.48	0.68	1.37	2.01	0.78	0.89
	120	251.49	0.75	1.47	1.97	0.70	0.90
	130	312.83	0.90	1.86	2.07	0.62	1.05
	140	286.47	0.98	1.98	2.02	0.65	0.96
	150	279.51	0.90	1.79	1.99	0.68	0.95
	160	69.26	1.00	2.16	2.16	0.30	0.43
Bedrock		31.53	0.66	1.13	1.71	0.27	0.31
T2	0	204.01	0.94	1.72	1.83	0.91	0.63
	5	181.34	0.87	1.43	1.65	0.84	0.62
	10	186.14	0.91	1.65	1.81	0.88	0.61
	15	188.69	0.85	1.50	1.77	0.87	0.62
	20	184.83	0.86	1.56	1.82	0.89	0.60
	30	182.73	0.84	1.54	1.83	0.89	0.61
	40	187.26	0.92	1.57	1.71	0.92	0.61
	50	173.41	0.91	1.64	1.80	0.85	0.61
	60	178.58	0.89	1.62	1.82	0.88	0.60
	70	188.74	0.89	1.47	1.66	0.94	0.61
	80	176.39	0.85	1.56	1.85	0.91	0.59
	90	186.60	0.87	1.52	1.75	0.93	0.61
	100	187.67	0.90	1.55	1.72	0.91	0.61
	110	188.34	0.84	1.52	1.82	1.02	0.62
	120	191.55	0.81	1.52	1.88	1.02	0.62
	130	200.43	0.78	1.43	1.85	1.10	0.62
T3	0	116.50	0.73	1.37	1.87	0.54	1.44
	5	116.27	0.73	1.28	1.76	0.51	1.78
	10	122.81	0.72	1.23	1.72	0.55	2.47
	15	104.96	0.75	1.31	1.76	0.48	2.34
	20	135.73	0.74	1.36	1.82	0.56	1.53
	30	138.35	0.78	1.32	1.69	0.45	2.80
	40	161.57	0.68	1.22	1.79	0.62	2.30
	50	184.77	0.70	1.26	1.79	0.71	2.58
	60	160.29	0.62	1.09	1.76	0.65	2.90
	70	153.70	0.65	1.17	1.81	0.56	2.32
Bedrock		30.17	0.71	1.23	1.72	0.25	0.34

## Appendix A

**Table A1:** REE concentrations and some characteristic parameters of samples in three soil profiles.

Sampling Sites	Depth (cm)	Fe (g·kg^−1^)	Mn (g·kg^−1^)	pH	Clay (%)	La (mg·kg^−1^)	Ce (mg·kg^−1^)	Pr (mg·kg^−1^)	Nd (mg·kg^−1^)	Sm (mg·kg^−1^)	Eu (mg·kg^−1^)	Gd (mg·kg^−1^)	Tb (mg·kg^−1^)	Dy (mg·kg^−1^)	Ho (mg·kg^−1^)	Er (mg·kg^−1^)	Tm (mg·kg^−1^)	Yb (mg·kg^−1^)	Lu (mg·kg^−1^)
T1	0.00	36.70	0.68	7.21	13.58	47.56	109.85	10.05	35.03	7.70	1.40	8.02	0.93	5.02	0.99	3.04	0.41	2.73	0.41
	5.00	37.20	0.64	7.68	14.34	39.71	82.35	8.21	29.12	6.17	1.14	6.47	0.76	4.19	0.85	2.57	0.37	2.43	0.37
	10.00	38.56	0.69	7.68	13.38	47.90	105.21	9.50	33.54	7.05	1.27	7.19	0.82	4.39	0.85	2.56	0.36	2.40	0.36
	15.00	36.72	0.67	7.70	14.68	42.03	97.58	8.65	30.25	6.63	1.27	6.58	0.79	4.22	0.84	2.49	0.34	2.26	0.35
	20.00	33.54	0.63	7.72	12.92	15.64	31.12	3.21	12.66	2.64	0.56	2.78	0.35	2.00	0.42	1.33	0.21	1.42	0.23
	30.00	34.20	0.61	7.72	14.41	36.73	84.66	7.63	26.76	5.85	1.17	5.89	0.68	3.72	0.75	2.22	0.30	2.05	0.31
	40.00	34.46	0.58	7.89	15.41	32.99	76.21	6.88	24.10	5.45	1.05	5.42	0.63	3.36	0.66	2.02	0.28	1.94	0.29
	50.00	29.73	0.56	7.85	13.99	35.83	70.64	7.39	26.73	5.69	1.12	5.70	0.68	3.64	0.70	2.14	0.29	1.92	0.29
	60.00	35.46	0.65	7.82	14.09	40.55	95.68	8.30	28.80	6.44	1.26	6.57	0.75	3.90	0.78	2.38	0.32	2.13	0.33
	70.00	24.00	0.45	7.50	15.99	40.77	93.93	8.34	29.02	6.47	1.29	6.54	0.77	4.21	0.83	2.47	0.34	2.25	0.34
	80.00	23.92	0.40	7.67	14.55	32.22	72.74	6.90	24.08	5.33	1.06	5.26	0.64	3.53	0.69	2.09	0.29	1.94	0.29
	90.00	30.42	0.34	7.50	12.38	43.58	99.99	9.32	33.72	7.50	1.49	7.56	0.92	4.97	0.99	2.94	0.40	2.66	0.40
	100.00	29.09	0.40	7.31	12.60	45.35	100.19	9.77	34.36	7.76	1.54	8.04	0.96	5.17	1.01	3.01	0.41	2.63	0.39
	110.00	25.64	0.42	7.28	12.66	42.96	95.57	9.25	33.07	7.27	1.42	7.36	0.88	4.75	0.93	2.75	0.37	2.43	0.36
	120.00	27.74	0.45	7.17	11.83	50.10	100.11	11.19	40.06	8.83	1.79	8.96	1.05	5.56	1.08	3.22	0.42	2.69	0.41
	130.00	29.49	0.43	7.06	11.00	59.26	96.06	12.78	46.30	9.70	1.91	9.65	1.19	6.09	1.18	3.51	0.46	2.97	0.44
	140.00	35.20	0.48	7.12	10.56	89.38	99.94	16.01	60.66	12.44	2.50	12.17	1.51	7.73	1.48	4.34	0.57	3.55	0.54
	150.00	33.92	0.51	7.21	9.07	82.83	100.00	13.77	50.33	10.41	2.10	10.34	1.25	6.53	1.25	3.62	0.49	3.09	0.46
	160.00	31.50	0.37	7.27	8.71	77.02	101.17	13.12	48.08	10.40	2.07	10.45	1.27	6.69	1.30	3.78	0.50	3.18	0.47
Bedrock		4.47	0.09			6.64	12.61	1.50	5.89	1.24	0.24	1.23	0.15	0.82	0.16	0.49	0.07	0.43	0.07
T2	0.00	22.13	0.30	4.82	10.12	43.27	104.60	8.34	27.45	5.53	1.01	5.61	0.57	2.88	0.57	1.79	0.26	1.86	0.29
	5.00	23.00	0.26	4.84	15.16	37.38	90.62	7.59	25.57	5.15	0.96	5.27	0.59	3.12	0.62	1.96	0.29	1.93	0.31
	10.00	23.49	0.28	4.88	14.05	38.22	95.22	7.66	25.80	5.16	0.93	5.12	0.56	2.88	0.56	1.77	0.26	1.71	0.28
	15.00	24.61	0.36	4.80	12.94	38.85	95.08	7.76	26.43	5.33	0.98	5.57	0.58	3.12	0.61	1.88	0.28	1.91	0.31
	20.00	23.14	0.33	5.01	14.10	37.47	96.15	7.45	24.48	5.02	0.93	5.34	0.54	2.84	0.56	1.73	0.26	1.78	0.28
	30.00	23.37	0.32	5.05	14.30	37.15	95.22	7.33	23.47	4.90	0.94	5.41	0.56	2.96	0.58	1.86	0.27	1.78	0.29
	40.00	23.88	0.33	5.07	16.12	37.69	98.85	7.41	24.61	4.94	0.92	4.98	0.53	2.76	0.54	1.72	0.26	1.77	0.28
	50.00	22.50	0.32	5.02	16.26	35.73	89.09	7.09	23.82	4.72	0.89	4.77	0.51	2.59	0.50	1.60	0.24	1.61	0.25
	60.00	22.16	0.31	5.08	17.90	35.94	93.22	7.23	24.07	4.87	0.90	4.92	0.51	2.63	0.51	1.63	0.24	1.63	0.27
	70.00	22.40	0.34	4.82	16.23	37.14	100.20	7.41	24.33	5.07	0.94	5.11	0.58	3.06	0.61	1.85	0.28	1.86	0.30
	80.00	21.92	0.31	4.78	17.04	34.63	94.45	6.97	22.23	4.67	0.88	5.00	0.52	2.70	0.53	1.67	0.25	1.64	0.26
	90.00	24.64	0.33	4.86	17.40	36.78	99.46	7.29	24.08	4.84	0.92	5.16	0.55	2.83	0.57	1.79	0.26	1.79	0.28
	100.00	22.27	0.32	4.87	16.37	37.81	97.95	7.47	24.97	4.99	0.93	5.12	0.57	3.01	0.60	1.89	0.28	1.80	0.29
	110.00	29.41	1.63	5.23	14.74	34.93	105.27	6.86	22.70	4.81	0.94	5.10	0.53	2.77	0.54	1.68	0.25	1.70	0.28
	120.00	31.47	0.78	5.06	17.82	35.49	106.85	6.95	23.34	4.77	0.95	5.35	0.53	2.77	0.56	1.72	0.26	1.72	0.28
	130.00	33.02	0.53	5.09	17.47	35.98	115.35	7.06	22.13	4.82	0.97	5.66	0.57	3.00	0.60	1.86	0.28	1.85	0.30
T3	0.00	14.58	0.22		0.00	24.70	47.57	5.22	19.76	6.10	2.20	4.12	0.50	2.57	0.49	1.48	0.21	1.33	0.27
	5.00	14.56	0.21	6.25	10.45	24.37	45.03	5.16	20.92	6.84	2.79	4.09	0.49	2.59	0.51	1.49	0.22	1.41	0.36
	10.00	17.08	0.23	6.31	10.79	24.20	47.92	5.15	20.93	9.12	4.24	4.11	0.50	2.61	0.51	1.48	0.22	1.45	0.36
	15.00	12.80	0.16	6.43	12.16	21.69	39.44	4.47	17.92	7.96	3.75	3.54	0.43	2.23	0.43	1.32	0.20	1.22	0.37
	20.00	15.78	0.19	6.53	12.13	28.99	53.45	6.01	24.60	7.47	2.54	4.75	0.57	2.95	0.57	1.70	0.25	1.58	0.31
	30.00	18.63	0.22	6.60	13.33	30.56	44.12	6.51	25.86	12.41	5.46	4.76	0.60	3.15	0.61	1.81	0.28	1.70	0.53
	40.00	22.84	0.25	6.77	13.30	32.39	63.99	7.38	26.85	10.60	4.41	5.82	0.71	3.75	0.73	2.18	0.32	1.97	0.48
	50.00	26.14	0.24	6.98	12.87	36.25	76.77	7.98	28.61	12.82	5.34	6.30	0.76	3.96	0.76	2.21	0.34	2.13	0.53
	60.00	27.93	0.26	6.99	11.36	29.23	63.93	6.86	25.28	13.01	5.94	5.77	0.72	3.80	0.73	2.13	0.33	1.98	0.58
	70.00	27.02	0.28	7.02	12.39	31.33	56.72	6.96	27.64	10.50	4.45	5.88	0.72	3.83	0.76	2.20	0.32	1.97	0.43
Bedrock		3.76	0.06			6.84	11.49	1.45	5.69	1.16	0.25	1.17	0.15	0.81	0.16	0.47	0.06	0.41	0.06
